# Metabolomic profiling of amines in sepsis predicts changes in NOS canonical pathways

**DOI:** 10.1371/journal.pone.0183025

**Published:** 2017-08-15

**Authors:** Abel Tesfai, Niall MacCallum, Nicholas S. Kirkby, Hime Gashaw, Nicola Gray, Elizabeth Want, Gregory J. Quinlan, Sharon Mumby, James M. Leiper, Mark Paul-Clark, Blerina Ahmetaj-Shala, Jane A. Mitchell

**Affiliations:** 1 Cardiothoracic Pharmacology, Vascular Biology, National Heart and Lung Institute, Imperial College London, London, United Kingdom; 2 Critical Care, University College London Hospital, London, United Kingdom; formerly critical care, National Heart and Lung Institute, Imperial College London, United Kingdom; 3 Department of Surgery and Cancer, Imperial College London, London, United Kingdom; 4 Respiratory, Airway Disease, National Heart and Lung Institute, Imperial College London, London, United Kingdom; 5 MRC Clinical Sciences, Imperial College London, Hammersmith Hospital, London, United Kingdom; Boston University School of Medicine, UNITED STATES

## Abstract

**Rationale:**

Nitric oxide synthase (NOS) is a biomarker/target in sepsis. NOS activity is driven by amino acids, which cycle to regulate the substrate L-arginine in parallel with cycles which regulate the endogenous inhibitors ADMA and L-NMMA. The relationship between amines and the consequence of plasma changes on iNOS activity in early sepsis is not known.

**Objective:**

Our objective was to apply a metabolomics approach to determine the influence of sepsis on a full array of amines and what consequence these changes may have on predicted iNOS activity.

**Methods and measurements:**

34 amino acids were measured using ultra purification mass spectrometry in the plasma of septic patients (n = 38) taken at the time of diagnosis and 24–72 hours post diagnosis and of healthy volunteers (n = 21). L-arginine and methylarginines were measured using liquid-chromatography mass spectrometry and ELISA. A top down approach was also taken to examine the most changed metabolic pathways by Ingenuity Pathway Analysis. The iNOS supporting capacity of plasma was determined using a mouse macrophage cell-based bioassay.

**Main results:**

Of all the amines measured 22, including L-arginine and ADMA, displayed significant differences in samples from patients with sepsis. The functional consequence of increased ADMA and decreased L-arginine in context of all cumulative metabolic changes in plasma resulted in reduced iNOS supporting activity associated with sepsis.

**Conclusions:**

In early sepsis profound changes in amine levels were defined by dominant changes in the iNOS canonical pathway resulting in functionally meaningful changes in the ability of plasma to regulate iNOS activity *ex vivo*.

## Introduction

Sepsis kills as many as 40% of patients and there are no drugs available that reduce mortality. The prevailing view is that vascular dysfunction and ultimately organ failure in sepsis is driven by excessive amounts of nitric oxide (NO) formed by the enzyme inducible nitric oxide synthase (iNOS). iNOS is expressed in leukocytes in response to invading pathogens as an essential part of the innate immune response. It is thought however, that in sepsis iNOS is also expressed in vascular [1–3] and cardiac cells leading to vasoplegia [1–3] and reduced cardiac function [4]. The amount of NO formed by iNOS is controlled by (i) levels of the substrate, L-arginine and (ii) levels of the substrate inhibitors asymmetric dimethylarginine (ADMA) and monomethylarginine (L-NMMA)[5]. ADMA and L-NMMA, together with symmetric dimethylarginine (SDMA) are known as ‘methylarginines’. Moreover, there are inherent complexities in the iNOS system because L-arginine cycles with other amino acids including L-citrulline, L-ornithine and L-glutamine [6]. This point is particularly relevant since levels of most amino acids are known to be altered in sepsis [7].

iNOS was first identified as a therapeutic target for sepsis in the early 1990s in a clinical case study that showed blocking NOS with L-NMMA reversed hypotension in two terminally ill patients with septic shock [8]. This, together with a substantial body of preclinical work, led to a large multiple-center clinical trial to assess the effects of L-NMMA on mortality. However, L-NMMA increased mortality and the trial was stopped prematurely [9]. It is thought that exogenous L-NMMA failed to deliver the promised therapeutic benefits because it acts in a non-discriminatory manner leading to ‘over correction’ of the NO pathway leaving the heart [10, 11] and other organs unprotected from the surge in endogenous constrictor hormones released during sepsis. As an alternative approach to administration of exogenous inhibitors it has been suggested that manipulating levels of endogenous levels of L-arginine, other amino acids and/or methylarginines could be a safer strategy to target iNOS activity in sepsis [12].

Since L-NMMA and ADMA are substrate inhibitors levels of L-arginine dictate their effect on iNOS. L-arginine is a semi-essential amino acid. This means that in healthy adults sufficient levels are generated endogenously [13–15], whereas in infants or in people with severe disease, including those with sepsis, L-arginine is not replete and must be supplemented from external sources [16]. Sepsis is associated with increased levels of ADMA [17, 18] and reduced levels of L-arginine [19, 20]. However, the precise relationship between methylarginines, arginine and other amino acids in the NO cycle in sepsis is not known. Moreover, the functional significance of sepsis related changes in plasma on iNOS activity and how this might be linked to arginine and ADMA has not yet been addressed. Thus, in the current study we have adopted a metabolomics approach to systematically measure a comprehensive array of amines, including amino acids and methylarginines in the plasma of a well-defined cohort of patients with sepsis. We have done this in order to determine the ***actual*** and ***relative*** importance of changes in NOS canonical pathways in sepsis to ultimately translate our findings to a personalised medicine approach for nutritional support in sepsis. Moreover, in order to interpret and biologically validate our findings, we developed a cell-based bioassay to test the functional significance of changes in plasma composition in sepsis on iNOS activity.

## Methodology

### Cell culture

RAW 264.7 mouse macrophages (ATCC, USA), were cultured using Dulbecco’s Modified Eagle’s Medium (Sigma-Aldrich, UK) supplemented with 2mM L-glutamine (Sigma Aldrich, UK), nonessential amino acids (Invitrogen, UK) and penicillin-streptomycin (Sigma Aldrich, UK) at 5% CO_2_ and 37°C. At confluence, cells were scraped and spun at 400 relative centrifugal force for 5 minutes. 10% filtered foetal bovine serum (LabTech, UK) was included only when culturing.

### Clinical study

Plasma samples were collected into anticoagulant heparin vacutainers from healthy volunteers (n = 21; 11M and 10F; 32.5 ± 6.7years) or sepsis patients (n = 38) at diagnosis (0) and 24 hours and 72 hours post diagnosis in the intensive care unit (ICU) at Royal Brompton Hospital, UK. Sepsis patient demographics are shown in Table 1. This study was approved by a Research Ethics Committee at Royal Brompton Hospital (RBH 01–152), Imperial College London (RBH 00–062). All volunteers gave written informed consent before entering the study.

**Table 1:** Patient demographics. Basic clinical information of patients with sepsis involved in the study is shown. Abbreviations for clinical assessments are APACHE II = Acute Physiology and Chronic Health Evaluation and SOFA = Sequential Organ Failure Assessment score. Data are shown as individual numbers for n = 38 for patients with sepsis.

Category	Number of patients (number/ mean)
Gender-M/F	M (22) and F (16)
Age (years)	60 ± 16.7
Body mass index (Kg/m^2^)	26.3 ± 0.7
Length of stay in ICU (days)	39.0 ± 5.4
Liver function tests	Good/normal (20) Moderate (8), Moderate dysfunction/poor (6) Severe dysfunction (3)
Right ventricular function	Good/normal (5) Moderate (5), Moderate dysfunction/poor (13) Severe dysfunction (1)
Clinical assessment; APACHE II SOFA 1–3	APACHE II: 18.7 ± 1.0 SOFA1: 9.2 ± 0.4 SOFA2: 9.0 ± 0.5 SOFA3: 8.5 ± 0.6
Diagnoses (more than one may be applicable per patient)	Sepsis/systemic inflammatory response syndrome/ multiple organ dysfunction (38) Pneumonia/acute respiratory distress syndrome (20) Endocarditis (1) Cardiac dysfunction (7) Acute renal failure (15)
Co-morbidities	Ischaemic heart disease (13) Hypertension (10) Non-insulin dependent diabetes mellitus (5) Asthma/chronic obstructive pulmonary disease (3) Peripheral vascular disease (4)
Mortality (%)	29.0 (6M and 5F)

### Measurements of iNOS supporting capacity of clinical plasma samples

Neat (100%) plasma was added directly to RAW 264.7 (1x10^5^) cells in the presence of LPS (1μg/ml) in a 96 well plate for 24 hours then used for nitrite measurements.

### Nitrite measurements

Samples were measured at 570nm by spectrophotometer (Infinite®F50; Tecan, Switzerland) using the Griess assay and a sodium nitrite standard curve (0-1mM).

### LC-MS/MS measurements

Samples were analysed as described previously [21] using an Agilent 6400 series triple quadruple liquid-chromatography tandem mass-spectrometry (LC-MS/MS) and mobile phase (0.1% formic acid, 1% acetonitrile). Read outs were from MassHunter Qualitative Analysis software (Agilent Technologies; Santa Clara, CA, USA) and concentrations from standard curves.

### ADMA and L-arginine ELISA

ADMA and L-arginine levels in clinical samples were determined using an enzyme immunoassay (DLD Diagnostika, Germany), as described previously [22].

### Ultra high performance liquid chromatography—Mass spectrometry (UHPLC-MS/MS) analysis of amines

Proteins were removed from plasma samples by precipitation with isopropanol (0.1% formic acid). Amines were measured using a HSS T3 UHPLC column system (Waters, Milford, MA, USA) connected to a Xevo TQ-S tandem quadrupole mass spectrometer (Waters, Wilmslow, UK) [23, 24]. Isotopically labelled standards (10μg/ml) and calibration curves (Sigma; 1–400μM) were used for quantification. MS/MS detection was via electrospray ionisation (ESI) in positive ion mode using multiple reaction monitoring (MRM) transitions. Analyte levels (reported as μM values) were calculated as a ratio of analyte peak area against appropriate internal standards, after 7.5 minute run.

### Heat map construction

UHPLC-MS/MS amine measurements from patients with sepsis were normalised to those from healthy patients and fold changes shown by pictographic scale representing a five-fold increase (red) or decrease (green).

### Ingenuity Pathway Analysis (IPA)

*A* ratio of healthy donor and patient amine measurements were analysed (IPA, Qiagen Redwood City, www.qiagen.com/ingenuity). The association between analytes and canonical pathways was tested by the Benjamini-Hocberg test with a false discovery rate of 0.05.

### Statistical analysis

Data are mean ± S.E.M for n donors/ experiments. Unless stated otherwise, all statistical tests were performed using GraphPad Prism v5 (GraphPad Inc., UK) and defined in figure legends. Statistical significance was noted when *p<0.05.

## Results and discussion

**Fig 1:**
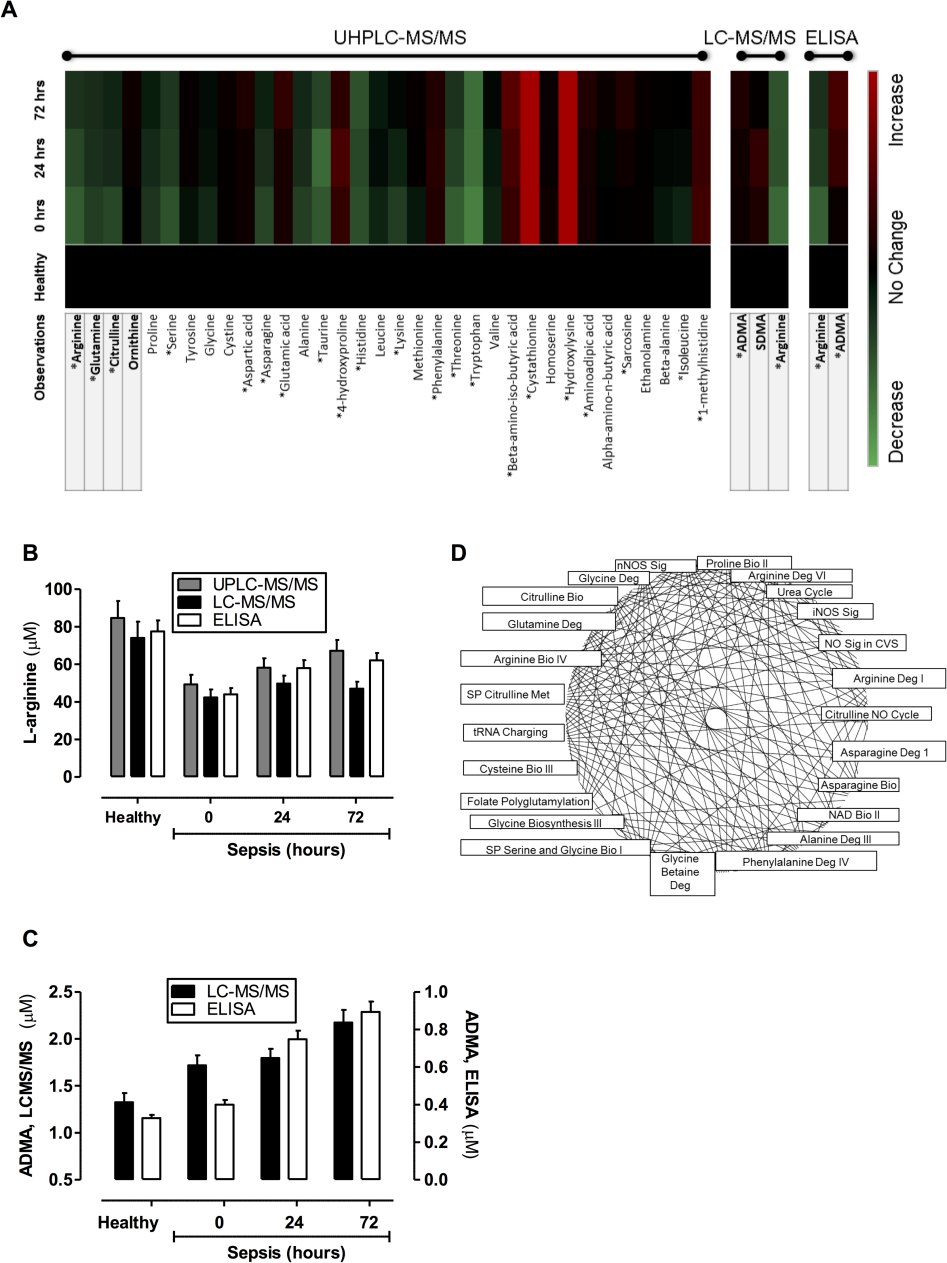
Targeted metabolic profiling of amines and methylarginines in human plasma from healthy donors and patients with sepsis. Amine and methylarginine levels were measured using **(A)** UHPLC-MS/MS, LC-MS/MS and/or ELISA in the plasma of healthy donors and patients with sepsis at diagnosis (0 hours), 24 hours and 72 hours post diagnosis. Comparisons between levels of L-arginine from **(B)** UHPLC-MS/MS, LC-MS/MS and ELISA and **(C)** ADMA from LC-MS/MS and ELISA are shown at diagnosis (0 hours), 24 hours and 72 hours, post diagnosis. **(D)** Canonical pathways extracted from IPA software were based on an input of read outs comparing plasma levels of amines in healthy donors and patients with sepsis as a ratio. Data are ± SEM for n = 21 healthy donors and n = 38 patients with sepsis. Data was analysed by one-way ANOVA with Dunnett’s post-hoc test and Benjamini-Hochberg test with a false discovery rate of 0.05; *p<0.05.

**Fig 2:**
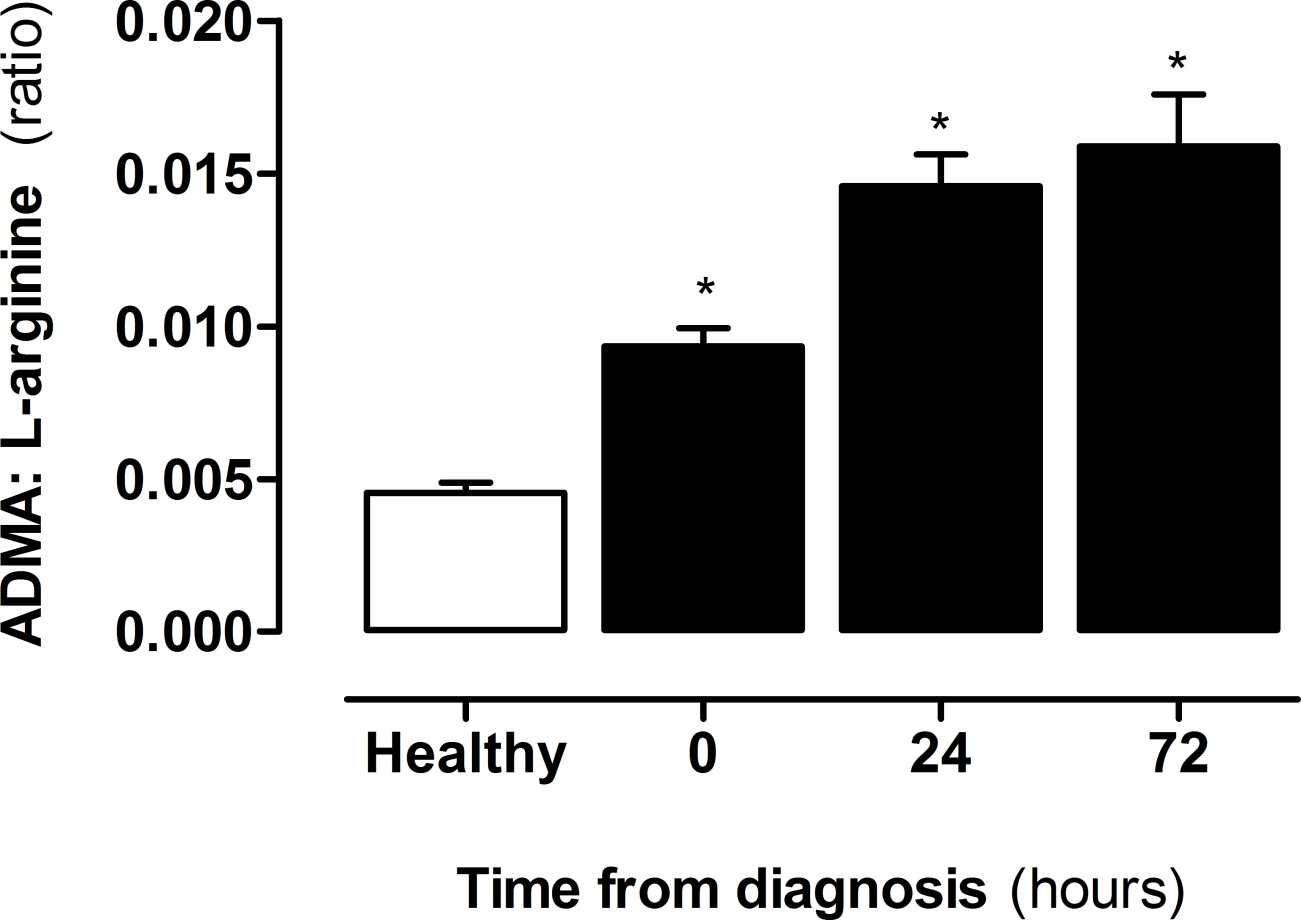
ADMA:L-arginine ratio in healthy donors vs patients with sepsis. ADMA and L-arginine were measured from plasma of healthy donors and patients with sepsis at diagnosis (0 hours), 24 and 72 hours post diagnosis using ELISA. Data are ± SEM for n = 21 healthy donors and n = 38 patients with sepsis. Data was analysed by one-way ANOVA with Dunnett’s post-hoc test. *p<0.05.

Three separate analytical techniques were used to determine the levels of amines in plasma from patients with sepsis. Firstly, an optimised UHPLC-MS/MS system was used to quantify 38 amines including L-arginine. Of those 38 analytes, 34 were measurable in human plasma and 21, including L-arginine, displayed significant differences in samples from patients with sepsis (Fig 1A; S1 Table). Secondly, to validate observations with L-arginine and to include the measurement of ADMA and SDMA, a second LCMS/MS approach was used (Fig 1B; S1 Table). Finally, for further validation measurements of L-arginine and ADMA were performed using ELISA (Fig 1C). It is commonly held that sepsis is a state of arginine deficiency, although a recent study found that arginine was increased in early sepsis [25]. In line with the other studies, here we found that L-arginine levels were lower in plasma of patients with sepsis within the first 24 hours after diagnosis but increased in the subsequent 48–72 hours. In our samples L-arginine levels were remarkably similar and showed identical trends when measured using each of the three analytical approaches (Fig 1). By contrast to L-arginine, ADMA levels increased at the point of diagnosis of sepsis and continued to rise at 24 and 72 hours (Fig 1). Again, as with L-arginine, whilst the absolute concentrations recorded were different, the pattern of change in ADMA was consistent when measured using LCMS/MS or ELISA. The reduced L-arginine and increased ADMA translated to an increase in ADMA:L-arginine ratio in plasma of patients with sepsis, and driven by increased ADMA, were elevated throughout the entire period of collection (Fig 2).

Pathway analysis of the total data set revealed significant changes in a range of canonical pathways extracted from IPA software (Fig 1D; S1 Fig). All three NOS pathways were significantly changed by sepsis and of all pathways, iNOS was found to be the most down regulated (S1 Fig). This result is in line with increased ADMA, reduced levels of L-arginine and reduced amino acids involved in the de novo synthesis of L-arginine such as L-glutamine and L-citrulline [13–15]. Together these findings strongly suggest that the ability of plasma from patients with sepsis to support optimal iNOS activity could be reduced and that in this setting strategies, such as blocking DDAH [12], to increase ADMA would indeed have functional consequences. To test this idea, we used a well-characterised mouse macrophage bioassay where iNOS activity is induced by LPS.

**Fig 3:**
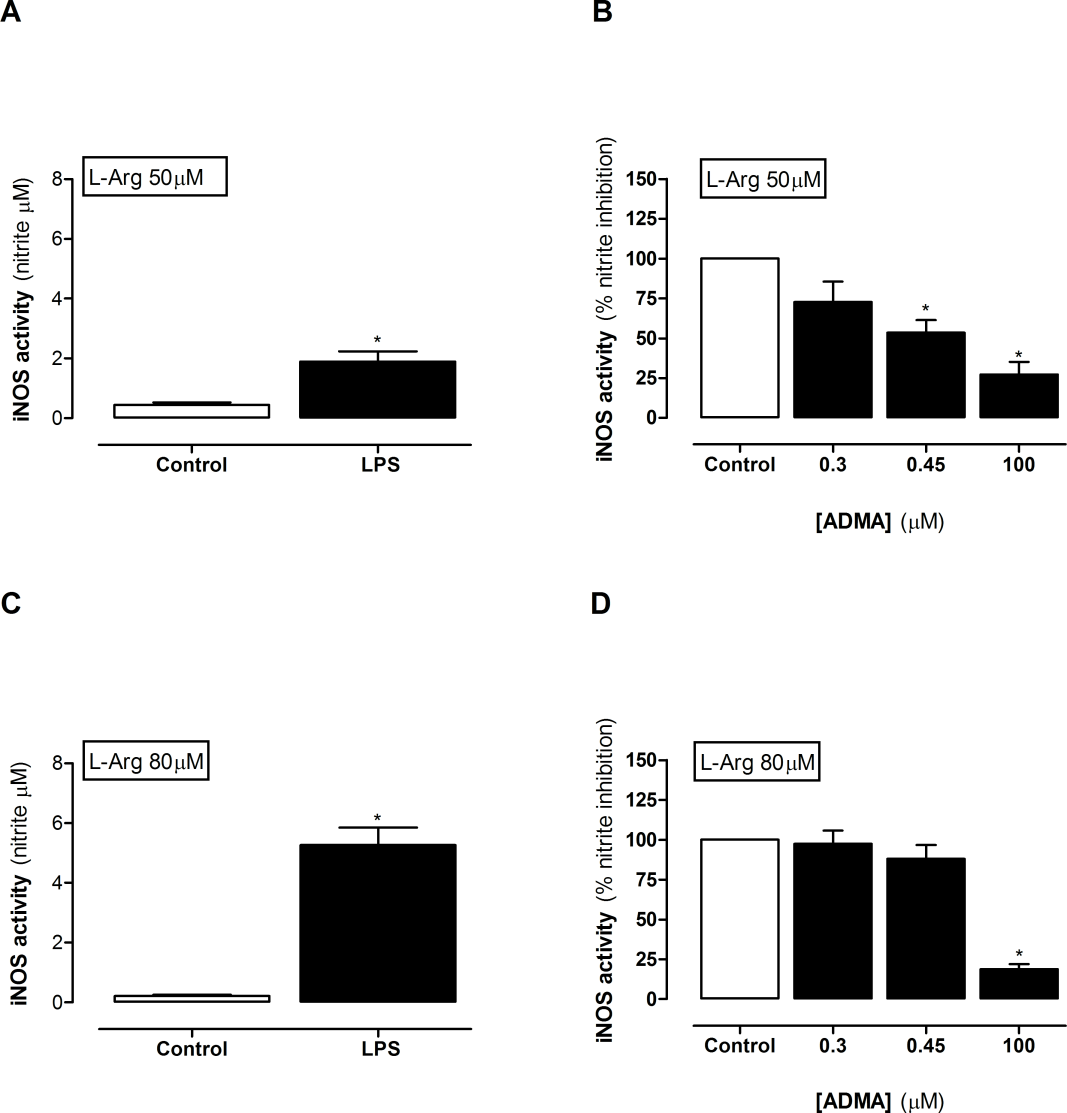
iNOS activity in LPS-activated mouse macrophages treated in culture media. iNOS activity in macrophages was measured in cells cultured in the presence of **(A,B)** 50μM or **(C,D)** 80μM L-arginine. Data are ± SEM for n = 4–6. Data was analysed by **(A,C)** paired t-test and **(B,D)** one-way ANOVA with Dunnett’s post-hoc test. *p<0.05.

**Fig 4:**
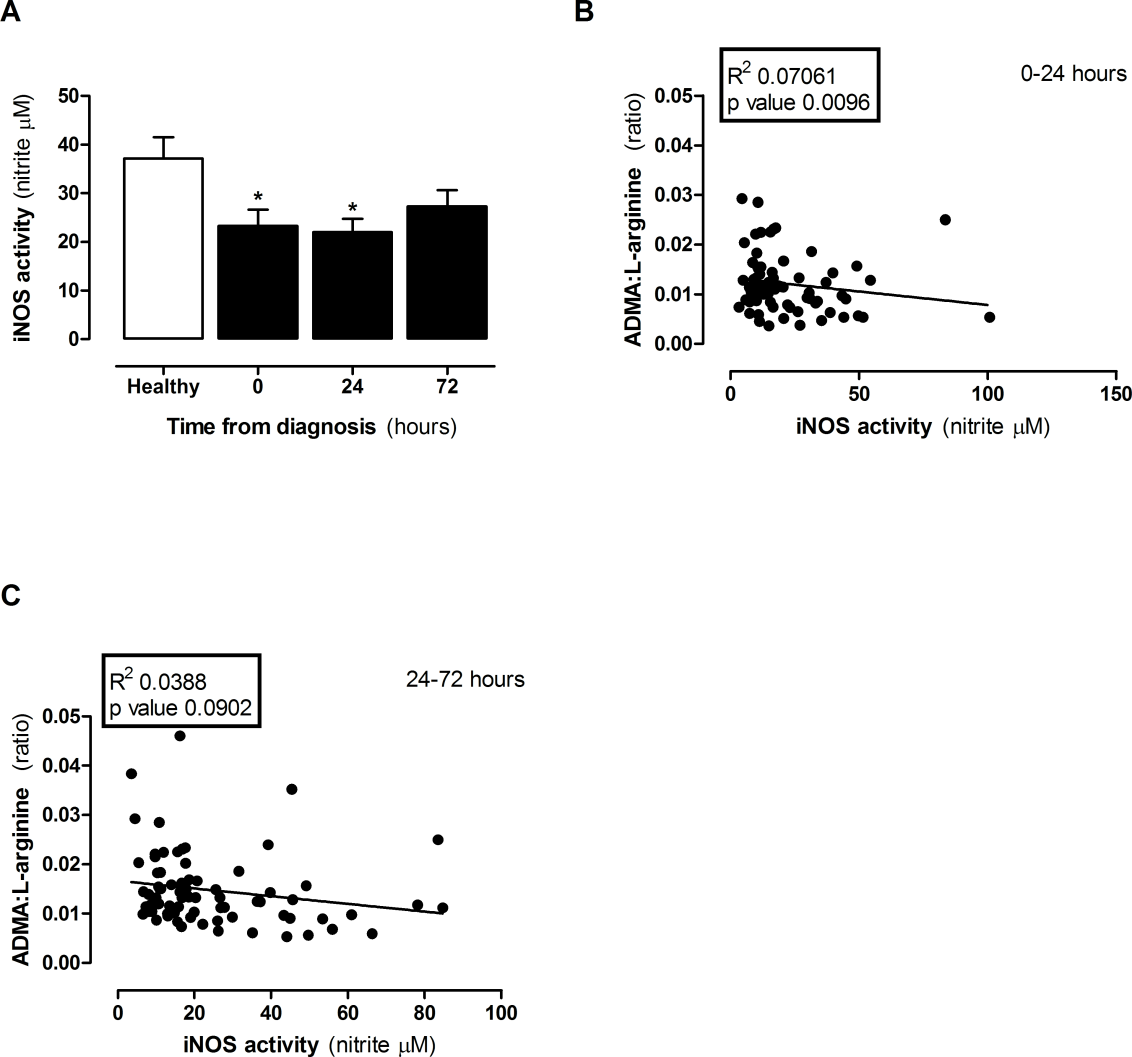
iNOS activity in LPS-activated mouse macrophages treated in plasma from healthy donors and patients with sepsis. **(A)** iNOS activity in plasma from healthy vs sepsis patients at 0, 24 and 72 hours. Correlations in ADMA:L-arginine ratio with iNOS activity at **(B)** 0–24 hours and **(C)** 24–72 hours post diagnosis. Data are mean ± SEM for n = 21 healthy donors and n = 38 patients with sepsis. Data was analysed by **(A)** Kruskal-Wallis one-way ANOVA with Dunn’s post-hoc test and **(B-C)** linear regression. *p<0.05 when compared to healthy.

When mouse macrophages are grown in regular culture media, which contains excess L-arginine (≈400μM), LPS induces concentration dependent increases in iNOS activity after 24 hours (S2A Fig). Under these conditions relatively high levels of ADMA are required to inhibit nitrite formation (S2B Fig). However, levels of L-arginine in human plasma are very much lower than those contained in regular culture media and, within the ranges we detected in this study, are rate limiting for iNOS activity (S2C Fig). In line with this we found higher levels of nitrite in media from LPS stimulated macrophages grown in media containing 80μM (eg healthy) than 50μM (e.g sepsis) L-arginine (Fig 3A and Fig 3C). Under these conditions the levels of ADMA present in plasma from patients with sepsis were sufficient to inhibit iNOS activity in the presence of 50μM (Fig 3B), but not in 80μM L-arginine (Fig 3D). These findings suggest that, assuming no other biological confounders were present; plasma from patients diagnosed with sepsis would have a reduced capacity to support iNOS activity. However, as >20 amino acids and amines were altered in the plasma of patients with sepsis, there may be other metabolic changes which overwhelm the effect of changed ADMA:L-arginine ratio on iNOS activity. To test this directly, plasma was added to LPS activated macrophages and iNOS activity measured. As predicted from the ADMA:L-arginine ratio, obtained from plasma of patients with sepsis, macrophages released lower levels of NO than those cultured in the presence of plasma from healthy control donors. This effect was seen in plasma taken from patients with sepsis at diagnosis and after 24hours but was lost in plasma from patients at 48–72 hours after sepsis had been diagnosed (Fig 4A). These findings were paralleled by a positive correlation between ADMA:L-arginine ratios and iNOS activity in cells within the first 24 hours (Fig 4B) but not after 24–72 hours (Fig 4C). These findings illustrate that in sepsis there is a global reduction in ‘iNOS supporting activity’ within the plasma. If this extrapolates to conditions within the body we would expect the entire NO pathway, whilst still active, to be compromised. The implications for this include (i) reduced endothelial derived NO which is consistent with reduced blood flow in some organs and (ii) reduced NO from immune cells attributing to immune suppression. Taken together these points support the idea of L-arginine supplementation in sepsis. However, iNOS is also expressed in vascular smooth muscle cells in sepsis where its activity is thought to underpin vasoplegia and associated pressure resistant hypotension. In this scenario L-arginine supplementation would enhance vascular dysfunction and could contribute to shock. Thus, the significance of a reduced capacity to support NO at the level of ADMA:L-arginine needs to be carefully considered in context of individual patients and requires further investigation before any impact on clinical management can be made.

In summary, there have been several studies in the literature showing that sepsis is associated with an increased ADMA:L-arginine ratio. However, ours is the first report that translates and validates observational measurements of ADMA and L-arginine to show meaningful biological consequences in sepsis conditions. This work provides essential proof of concept evidence for the idea that targeted metabolic profiling of amine levels in patients with sepsis can identify temporal nutritional deficiencies and to the notion that enhancing endogenous levels of ADMA, will impact on iNOS activity despite the complex changes in plasma constituents seen in sepsis. How this information may be harnessed to develop patient specific, personalised medicine, interventions remains the subject of investigation.

## Supporting information

S1 Fig List of the top 20 canonical pathways altered in plasma of patients with sepsis. A total of 134 pathways, of which 77 were significantly altered were determined (top 20 shown). Data are shown as the percentage and number of pathway molecules down or upregulated. Ratios were generated for each of 34 analytes using data for n = 21 healthy donors and n = 38 patients with sepsis. Data was analysed by Benjamini-Hochberg test with a false discovery rate of 0.05 applied. Individual P values are shown in brackets.
(TIF)

S2 Fig **Effect of (A) LPS and (B) ADMA in control media (contains ≈400**μ**M arginine) and (C) L-arginine in arginine free media on iNOS activity in mouse macrophages.** iNOS activity was determined from nitrite concentrations in conditioned media after 24 hours. LPS was added at 1μg/ml in panels **(B)** and **(C)**. Data are mean ± SEM and n = 3 individual experiments. Data was analysed by one-way ANOVA followed by Dunnett's post-hoc test compared to control. *p<0.05.
(TIF)

S1 Table List of amines measured in human plasma using UHPLC-MS/MS, LC-MS/MS and/or ELISA. Data are mean ± SEM for n = 21 healthy donors and n = 38 patients with sepsis. Data was analysed by either one-way ANOVA with Dunnett’s post-hoc test or, where appropriate, Kruskal-Wallis one-way ANOVA with Dunn’s post-hoc test. *p<0.05 when compared to healthy controls.
(TIF)
