# Supplementary figures and images for: Metabolomic profiling of amines in sepsis predicts changes in NOS canonical pathways
Source: PLoS One. 2017 Aug 15;12(8):e0183025. doi: 10.1371/journal.pone.0183025 (PMC5557592; doi:10.1371/journal.pone.0183025)

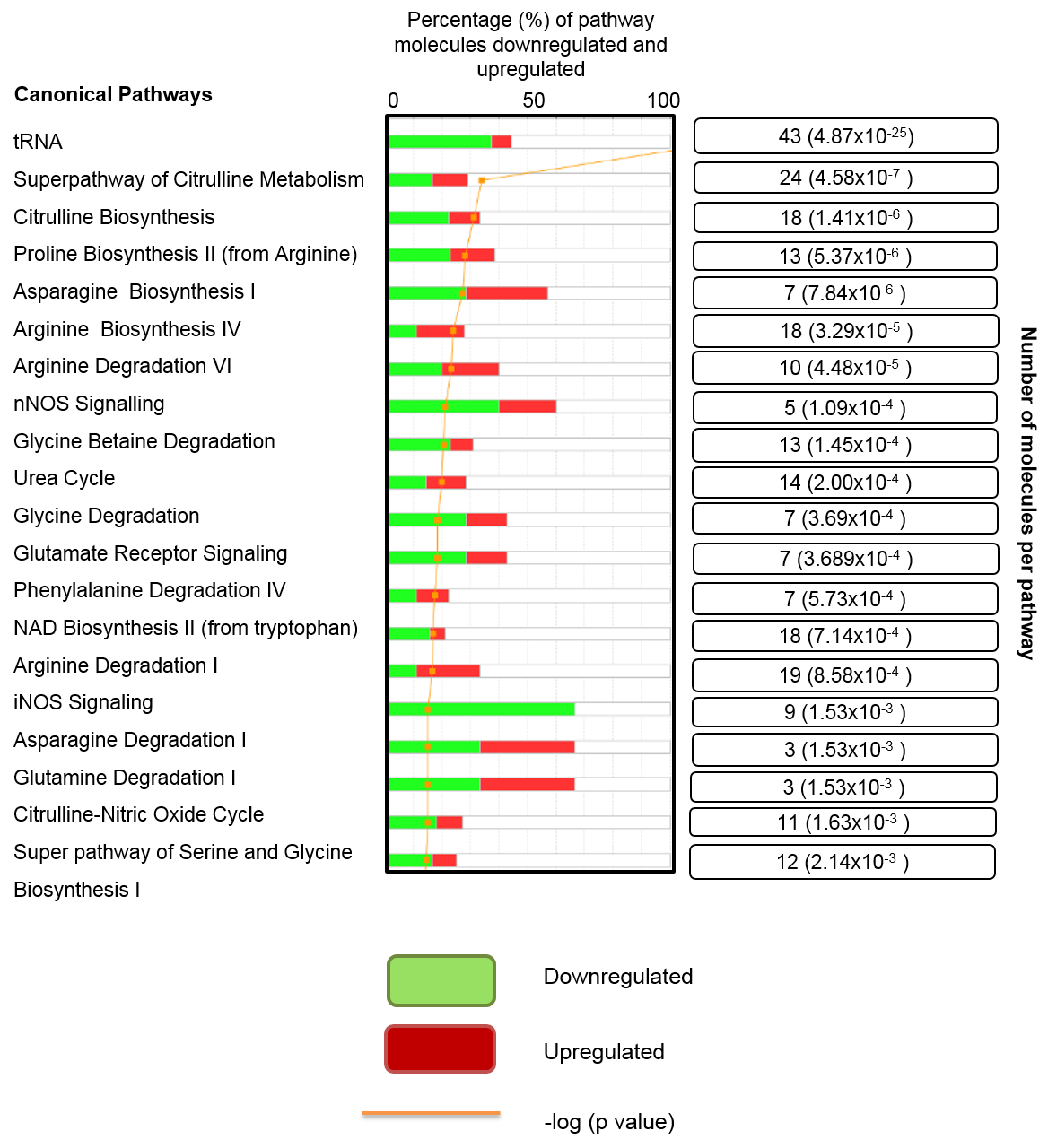

Supplement: S1 Fig — A total of 134 pathways, of which 77 were significantly altered were determined (top 20 shown). Data are shown as the percentage and number of pathway molecules down or upregulated. Ratios were generated for each of 34 analytes using data for n = 21 healthy donors and n = 38 patients with sepsis. Data was analysed by Benjamini-Hochberg test with a false discovery rate of 0.05 applied. Individual P values are shown in brackets. (TIF) [file pone.0183025.s001.tif]

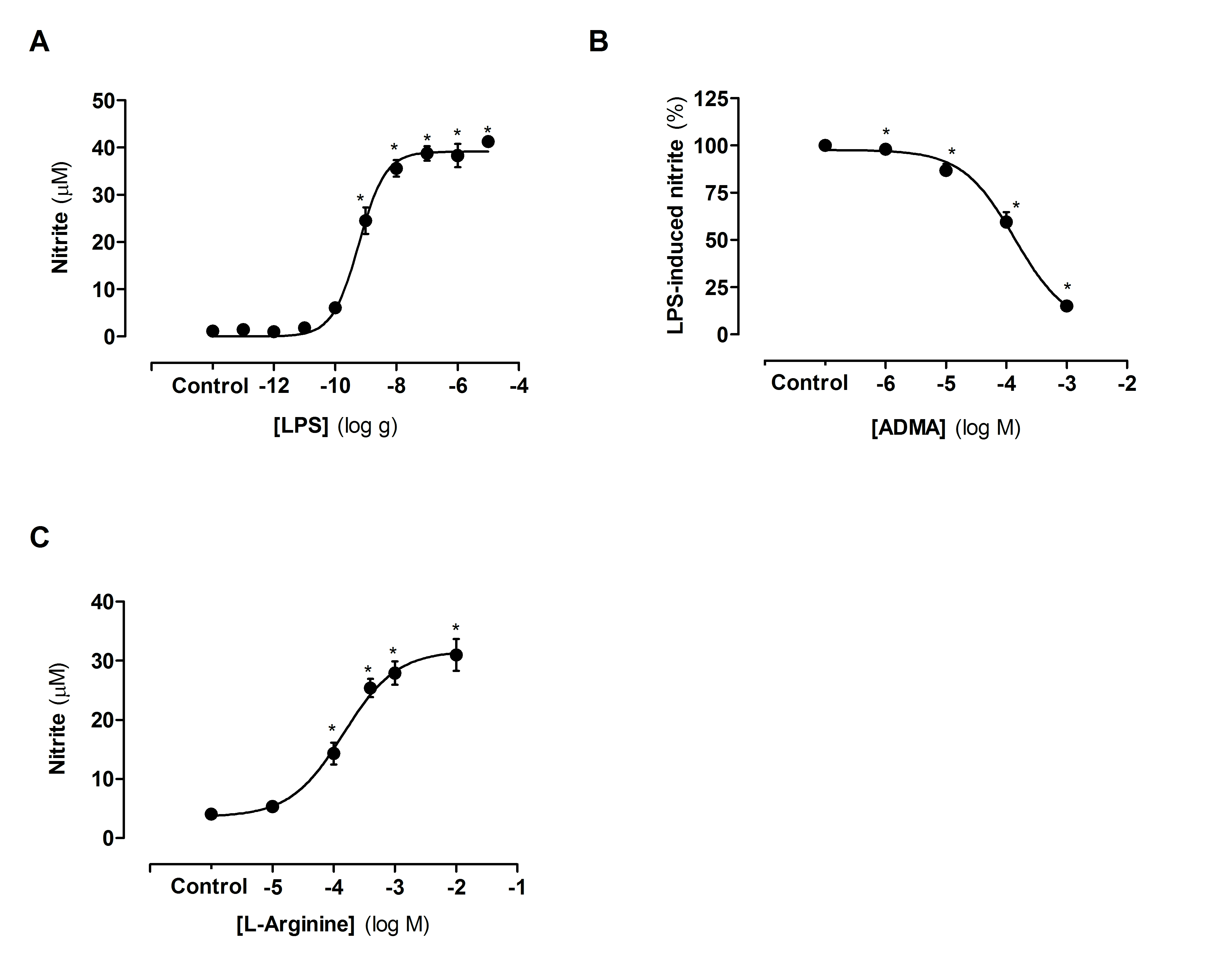

Supplement: S2 Fig — Effect of (A) LPS and (B) ADMA in control media (contains ≈400μM arginine) and (C) L-arginine in arginine free media on iNOS activity in mouse macrophages. iNOS activity was determined from nitrite concentrations in conditioned media after 24 hours. LPS was added at 1μg/ml in panels (B) and (C). Data are mean ± SEM and n = 3 individual experiments. Data was analysed by one-way ANOVA followed by Dunnett's post-hoc test compared to control. *p<0.05. (TIF) [file pone.0183025.s002.tif]

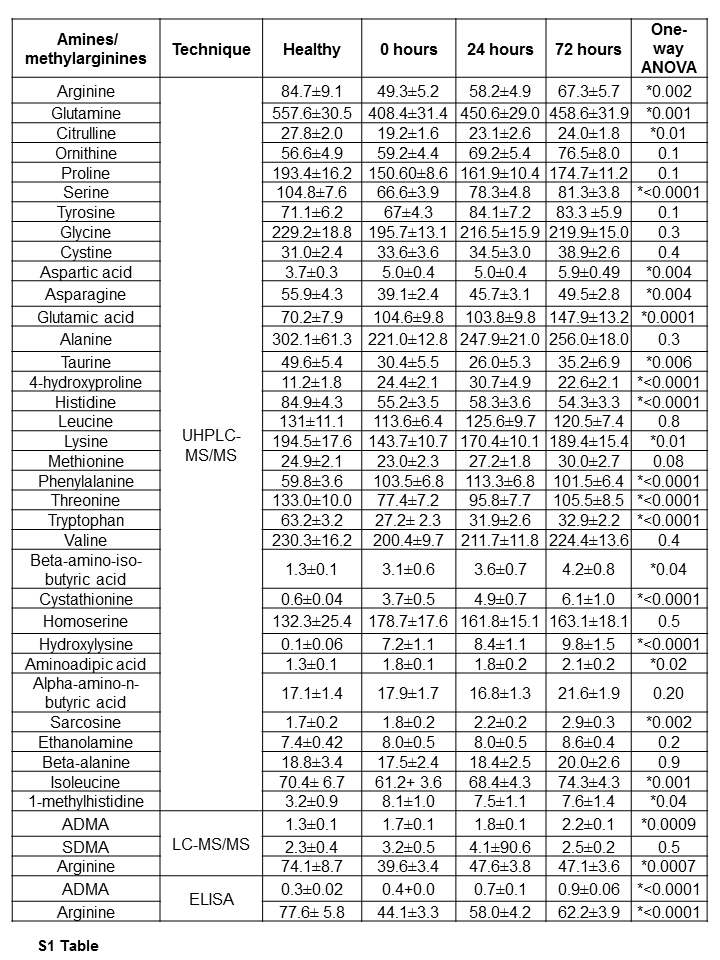

Supplement: S1 Table — Data are mean ± SEM for n = 21 healthy donors and n = 38 patients with sepsis. Data was analysed by either one-way ANOVA with Dunnett’s post-hoc test or, where appropriate, Kruskal-Wallis one-way ANOVA with Dunn’s post-hoc test. *p<0.05 when compared to healthy controls. (TIF) [file pone.0183025.s003.tif]
